# The Close Relationship between Sleep Disorders and Reproductive Dysfunction in Endocrinology

**DOI:** 10.3389/fendo.2012.00117

**Published:** 2012-10-15

**Authors:** Shailendra Kapoor

**Affiliations:** ^1^Mechanicsville, VA, USA

The recent article by Williams and Kriegsfeld (2012) was highly interesting. Notably, decreased reproductive function with co existent sleep disorders may be seen in a number of pathological conditions in endocrinology.

For example, patients with obstructive sleep apnea (OSA) usually demonstrate low levels of free testosterone. These decreased levels in OSA patients are remarkably independent of the body mass index. For instance, the mean free testosterone level in patients with severe OSA is about 69 pg/ml in comparison to 75 pg/ml in those without any OSA (Hammoud et al., 2011). In fact, a negative relationship exists between serum testosterone levels and the hypopnea index. Obesity also results in increased scrotal temperature that can contribute to infertility (Cabler et al., 2010). Nocturnal scrotal cooling is one treatment option in males with oligo-astheno-teratozoospermia.

Overall, a reduced quality of life is seen in males with OSA. Testosterone levels are improved following the successful treatment and management of OSA with Continuous Positive Airway Pressure (CPAP) therapy. Similar improvements in erectile dysfunction can be seen following successful treatment of OSA. A significant decline in prolactin levels is also noticed following the institution of CPAP therapy thus having a further positive impact on male fertility.

Sleep apnea may also be seen with decreased reproductive function and adjunct obesity in females. Such patients may have underlying polycystic ovarian syndrome (PCOS; Randeva et al., 2012), a condition seen in nearly 7% of all females of the reproductive age group (Dunaif, 2011). Anti-diabetic agents such as metformin have been used successfully for controlling the symptoms of PCOS. Similarly, spironolactone has shown considerable promise in the management of PCOS. An increased incidence of sleep apnea along with decreased reproductive function and obesity is also seen in patients with Down’s syndrome (Jensen et al., 2012). Obesity and decreased reproduction in adjunction with OSA may be seen in underlying achondroplasia (Pauli, 1993), an autosomal dominant disorder typically caused by a mutation in the FGFR3 gene. Patients with achondroplasia typically have hypotonic, short extremities with a comparatively large cranium. Typical facial features include mid-face retrusion and frontal bossing.

Individuals with acromegaly may have coexistent OSA and decreased reproductive function (Giustina et al., 2003). In fact, patients with acromegaly demonstrate a close relationship between soft tissue overgrowth in the larynx and OSA. Somatostatin analogs such as sandostatin decrease growth hormone levels and attenuate upper respiratory tract soft tissue overgrowth thus improving OSA symptoms in patients with acromegaly. Prader–Willi syndrome is another pathological condition in which obesity and infertility may co-exist with sleep disturbances. Daytime hypersomnolence is especially seen in patients with Prader–Willi syndrome. An increased incidence of OSA is also seen in Prader–Willi syndrome.

Females with irregular menstrual cycles have almost twice the incidence of sleep abnormalities such as “light sleep stages” in comparison to those with regular menstrual cycles (Hachul et al., 2010). Females with underlying fibromyalgia also have co existent decreased reproductive function in addition to a higher incidence of sleep abnormalities (Shaver et al., 2006).

The above examples clearly illustrate a higher incidence of reproductive dysfunction and sleep disorders in patients with the above conditions and the need to regularly screen such patients for sleep disorders.
